# Testosterone inhibits the growth of prostate cancer xenografts in nude mice

**DOI:** 10.1186/s12885-017-3569-x

**Published:** 2017-09-07

**Authors:** Weitao Song, Vikram Soni, Samit Soni, Mohit Khera

**Affiliations:** Scott Department of Urology, Baylor College of Medicine, Jones Building 506C, One Baylor Plaza, Houston, TX 77030 USA

**Keywords:** Prostate cancer, Androgen, Testosterone

## Abstract

**Background:**

Traditional beliefs of androgen’s stimulating effects on the growth of prostate cancer (PCa) have been challenged in recent years. Our previous in vitro study indicated that physiological normal levels of androgens inhibited the proliferation of PCa cells. In this in vivo study, the ability of testosterone (T) to inhibit PCa growth was assessed by testing the tumor incidence rate and tumor growth rate of PCa xenografts on nude mice.

**Methods:**

Different serum testosterone levels were manipulated in male nude/nude athymic mice by orchiectomy or inserting different dosages of T pellets subcutaneously. PCa cells were injected subcutaneously to nude mice and tumor incidence rate and tumor growth rate of PCa xenografts were tested.

**Results:**

The data demonstrated that low levels of serum T resulted in the highest PCa incidence rate (50%). This PCa incidence rate in mice with low T levels was significantly higher than that in mice treated with higher doses of T (24%, *P* < 0.01) and mice that underwent orchiectomy (8%, *P* < 0.001). Mice that had low serum T levels had the shortest tumor volume doubling time (112 h). This doubling time was significantly shorter than that in the high dose 5 mg T arm (158 h, *P* < 0.001) and in the orchiectomy arm (468 h, *P* < 0.001).

**Conclusion:**

These results indicated that low T levels are optimal for PCa cell growth. Castrate T levels, as seen after orchiectomy, are not sufficient to support PCa cell growth. Higher levels of serum T inhibited PCa cell growth.

## Background

Traditional beliefs of androgen’s stimulating effect on the growth of PCa have been challenged in recent years. Recent literature even suggests that men with lower serum T levels are more likely to have PCa. There is also emerging data to suggest that T may even be protective against PCa growth [1–9]. Our previous study indicated that physiological normal levels of androgen inhibit the proliferation of PCa cells in vitro, though low levels of androgen are essential for initial growth of PCa cells [10]. In this in vivo study, the ability of testosterone to inhibit PCa growth was assessed by testing the tumor incidence rate and tumor growth rate of PCa xenografts on nude mice with different serum T levels manipulated by orchiectomy or implanting different dosages of T pellets subcutaneously.

## Methods

### Cell culture and tumor xenografts development on nude mice

PCa LNCaP cells (ATCC, CRL-1740, clone FGC) were used to develop PCa tumor xenografts on mice. The cells were cultured in RPMI 1640 medium (Gibco, 61870036) supplemented with 10% fetal bovine serum (Hyclone, SH30910.03HI) and 1X Antibiotics-Antimycotic (Gibco, 15240062) in incubator (37 °C, 5% CO2 atmosphere). When developing tumor xenografts, cultured LNCaP cells (passages 20 to 50) were detached and separated by 0.25% trypsin-EDTA solution (Gibco, 25300) and washed by serum free medium. Five million cells in 200ul serum free medium were inoculated to each mouse with a 23 gauge needle subcutaneously between the shoulders. Male nude/nude athymic mice (Jackson Lab, 007850) aged 5 to 6 weeks (average weight 22.3 g, range 20.7-23.6) were used in the experiments.

### Tumor incidence rate study

The mice were divided and randomized into 4 arms. Mice either underwent orchiectomy or had implantation of a 2 mg or 5 mg T pellet (Testopel, Bartor Pharmacal). These mice were referred to as orchiectomy arm, 2 mg T arm, and 5 mg T arm, respectively. The forth arm consisted of control mice.

Each arm contained 50 mice for a total of 200 mice in this experiment. The sample size was calculated with nQuery on the basis of a small pilot experiment. Serum T levels were manipulated as above on five to six week-old mice. One week after T level manipulation, five million LNCaP cells were injected subcutaneously. Tumor incidence was observed every two to three days for 12 weeks. In order to record the data by an observer blinded to the androgen status of the mice, the mice were mixed in cages. Each cage had five mice and at least one mouse from each of the 4 arms. Tumor incidence rate of each arm was analyzed.

### Tumor growth rate study

An additional 160 mice at five to six week-old were injected subcutaneously with five million LNCaP cells and tumor development was observed every two to three days for 90 days. After the tumor size reached 3X3mm, the mice were divided into the four aforementioned arms. The serum T levels were manipulated by orchiectomy and T pellet implantation as described previously. A total of 75 mice were found to have palpable tumor and were then randomized into each arm of the growth rate experiments. Each arm contained 18 to 20 mice. After serum T level manipulation, tumor size was measured every two to three days until 14 weeks. The observer was also kept blinded to the androgen status of the mice. Tumor volume was calculated by the formula W^2^ X L/2. The average tumor size and tumor volume doubling time of each arm was analyzed.

### Mice serum T tests

Blood samples were collected from the mice tails. Time points for serum T assessment include before T manipulation surgery and then at 3 days, 1, 2, 3, 4, 5, and 6 weeks after T manipulation surgery. For each time point, 16 mice were randomly chosen from each arm in the tumor incidence rate study. Serum T levels were tested by Elisa Kit (Rocky Mountain Diagnostic, AA E-1300). Briefly, a duplicate 25ul of each calibrator, control, and serum from each sample were added to the wells of the ELISA plate and 200ul of the enzyme conjugate working solution was also added into each well. After 1 h of incubation at room temperature, the plate was washed three times and 200ul of TMB substrate was added into each well. Incubate the plate at room temperature for 15-20 min. Pipette 100ul of stop solution to each well and the plate was read within 10 min on a microplate reader at 450 nm. The raw data was analyzed with the software Prism 3.0.

### Ethics approval and consent to participate

All animal breeding, care, and experimentation procedures followed the guideline according to protocols approved by the Institutional Animal Care and Use Committee of Baylor College of Medicine and conformed to the *Guide for the care and use of laboratory animals* (NIH Publication no. 85-23. Revised 1996).

Mice were fed a standard mouse diet, and housed in standard mouse cages on a 12 h inverted light-dark cycle. All surgery procedures, cell injection, and tumor measurement were performed in special animal surgery site when mice got inhalational anesthesia with isoflurane vaporizer. When in need, the mice were euthanized with CO2 rodent euthanasia chamber.

In the tumor growth rate experiment, we designed the study to further observe mice caring tumors for 14 weeks after tumor size reached 3X3mm so that we could obtain ample data for comparing the growth rates. The tumor size was measured every 2 to 3 days by lab technician. When tumor size reached/exceed the limitation (2.0 cm in any dimension), the mice were euthanized. Many mice were euthanized in the planned experiment period, e.g. before 14 weeks since the tumor size reached/exceed the limitation, especially in control arm. Thus, we only obtained data for comparing the growth rate in the first month.

### Statistical analysis

The statistical significance of the tumor incidence rate in each arm was analyzed by Chi-Square test. In the tumor growth rate experiments, statistical significance of the tumor volume doubling time in each arm was compared with the student *t* test. Data were shown as mean ± s.d.

## Results

### Mice serum T level

**Fig. 1:**
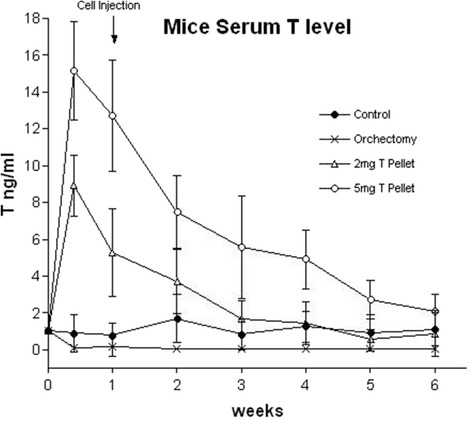
Graph shows nude mice serum T level changes inside 6 weeks by the manipulation of orchiectomy or different amount of T pellet implantation. Each time point has tested 16 samples from randomly chosen mice in each arm

Serum T levels of 5 to 11 week old control male nude mice were an average 1.0 ng/ml. The serum T level had no significant changes from the age 5 weeks to 11 weeks. Castrated mice’s serum T level was extremely low at an average 0.1 ng/ml. In the castrated mice, the serum T level had no significant change from 1 to 6 weeks after orchiectomy (age 6 weeks to 11 weeks). Two mg T pellet implantation resulted in mice’s serum T levels lasting 2.5 weeks above 2.4 ng/ml with the peak level at day 3 after implantation (The normal range of adult men’s serum T level is 2.4 to 9.5 ng/ml, Mayo Clinic). Five mg T pellet implantation resulted in mice’s serum T levels lasting 5 weeks above 2.4 ng/ml and the peak was also at day 3 after implantation (Fig. 1).

### Tumor incidence rate study

**Fig. 2:**
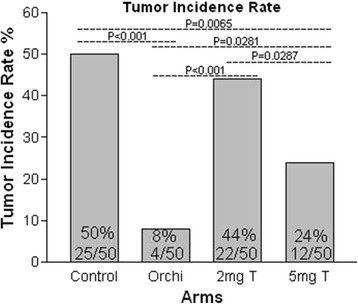
Tumor incidence rate was the lowest in orchiectomy arm and it was significantly lower than that in all the other three arms. The PCa incidence rate in the 5 mg T arm was also significantly lower than that in both control and 2 mg T arms. Data was analyzed by Chi-square test

The control arm had the highest tumor incidence rate (50%, 25/50), which was significantly higher than the rate in the 5 mg T arm (24%, 12/50, *P* < 0.01) and the orchiectomy arm (8%, 4/50, *P* < 0.001). There was no significant difference in the PCa tumor incidence rate between the control arm and the 2 mg T arm (44%, 22/50, *P* > 0.05). The orchiectomy arm had the lowest tumor incidence rate when compared to 5 mg T arm (*P* < 0.05), the 2 mg T arm (*P* < 0.001), and the control arm (*P* < 0.001). The tumor incidence rate in the 5 mg T arm was significantly lower than that in the 2 mg T arm (*P* < 0.05) (Fig. 2).

The presence of tumor in mice was checked every two to three days. Palpable tumor appeared between 16 and 75 days post-inoculation. Tumor was found between 17 and 72 days in the control arm (mean 42.5, SD 15.7), 35 and 72 days in the orchiectomy arm (mean 51.7, SD 18.8), 16 and 75 days in the 2 mg T arm (mean 38.0, SD 15.3), and 35 and 75 days in the 5 mg T arm (mean 46.5, SD 12.9). There were no significant differences between arms on average days the tumors appeared post-inoculation. However, none of the tumors appeared in less than 35 days after LNCaP cell injection in 5 mg T arms (0%, 0/12) when the serum T level was maintained above 2.4 ng/ml. In the control arm and 2 mg T arm, the percentage of tumor appearing in less than 35 days were 32% (8/25) and 45% (10/22), respectively. These ratios were significantly higher than that in the 5 mg T arm (*P* < 0.05).

The tumor incidence rate data indicated that the extremely low castrate androgen levels did not support PCa cells growth. PCa cells grew best in mice with serum androgens at low levels, while higher levels of androgens inhibited PCa cell growth.

### Tumor growth rate study

**Fig. 3:**
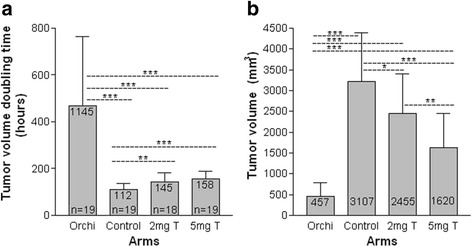
**a** Tumor growth rate was the lowest in the orchiectomy arm in the first month. The tumor volume doubling time was significantly longer than that in all the other three arms. The tumor volume doubling time in both the 2 mg T and 5 mg T arms were also significant longer than that in control arm. **b** Comparison of the average tumor size in each arm after one month. Data was analyzed by *t* test. *P < 0.05, ** *P* < 0.01, ***P < 0.001

The results of tumor growth rate experiments were analyzed by comparing the tumor volume doubling time and average tumor size in the first month in each arm. The orchiectomy arm had the lowest tumor growth rate with tumor volume doubling time of 467.9 h (range 209.4 to 1145.4, SD 296.4). This was significantly longer that that in 5 mg T arm (157.7 h, range 118.6 to 233.1, SD 32.2, *P* < 0.001), 2 mg T arm (144.5 h, range 87.0 to 238.3, SD 43.9, *P* < 0.001), and control arm (112.1 h, range 70.3 to169.3, SD 26.0, *P* < 0.001). The control arm had the highest growth rate with the tumor doubling time significantly shorter than that in 5 mg T arm (*P* < 0.001) and 2 mg T arm (*P* < 0.01) The average tumor size was the smallest in orchiectomy arm (457 mm^3^, SD 321), which was signifcantly smaller than that in control arm (3107 mm^3^, SD 1240 *P* < 0.001), 2 mg T arm (2455 mm^3^, SD 910, *P* < 0.001), and 5 mg T arm (1620 mm^3^, SD 828, *P* < 0.001). The average tumor size in control arm was larger than that in 2 mg T arm (*P* < 0.05) and 5 mg T arm (*P* < 0.001). And the average tumor size in 2 mg arm was also larger than that in 5 mg arm (*P* < 0.01) (Fig. 3). These findings were consistent with the results of the tumor incidence rate study which indicated that optimal PCa cell growth is at low androgen levels.

**Fig. 4:**
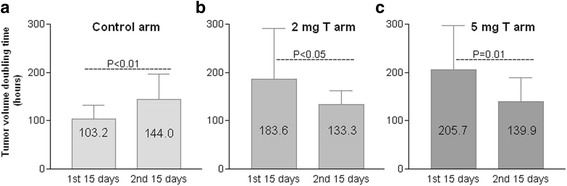
Comparison of tumor volume doubling time in control arm, 2 mg T arm, and 5 mg T arm in the first month after the tumor appeared and the serum T levels were manipulated. The serum T level in mice of the control arm maintained an average of 1 ng/ml throughout the month (Fig. 1) and the tumor volume doubling time became longer in the second 15 days than that in the first 15 days (**a**). The serum T levels in the 2 mg T arm were higher than 3.5 ng/ml in the first 15 days and then declined to lower than 2 ng/ml in the second 15 days. The serum T levels in the 5 mg T arm were higher than 7 ng/ml in the first 15 days and then declined to 4 ng/ml in the second 15 days (Fig. 1). The tumor volume doubling time became shorter in the second 15 days compared to the first 15 days in both 2 mg and 5 mg T arms (**b** and **c**)

In control arm, the tumor grew the fastest within the first couple weeks. As the tumor enlarged, we noticed a decline in growth rate of these tumors. In the control arm, the tumor growth rate was significantly higher in the first 15 days compared to the second 15 days. The tumor volume doubling time changed from 103 h in the first 15 days to 144 h in the second 15 days (*P* < 0.01). However, in the 2 mg T and 5 mg T arms, the opposite results were found. The data demonstrated that in 2 mg T and 5 mg T arms, the tumor growth rate was significantly lower in the first 15 days compared to the second 15 days, (184 h vs 133 h in 2 mg T arm, *P* < 0.05, 206 h vs 140 h in 5 mg T arm, *P* < 0.01). When comparing the growth rates of the control arm and the 2 mg T and 5 mg T arms in the first 15 days, the growth rates were much higher in the control arm (*P* < 0.01). However, in the second 15 days there was no significant difference in tumor growth rates in the control arm, 2 mg T arm, and 5 mg T arms (Fig. 4). This data indicated that higher serum T levels in the 2 mg T and 5 mg T arms in the first 15 days inhibited the PCa cell growth. The fastest growth period was during the second 15 days when serum T level went down. There was no significant difference in the growth rate in the control arm, 2 mg T arm, and 5 mg T arm in the second 15 days. When comparing the growth rates of the control arm, 2 mg T arm and 5 mg T arm in their fastest growth periods (first 15 days in control arm, second 15 days in 2 mg T and 5 mg T arms), it was found the rate in control arm was higher than that in 2 mg T arm (103 vs 133 h, *P* < 0.01) and 5 mg T arm (103 vs 140 h, *P* < 0.01). In the orchiectomy arm, most of the tumor did not grow initially for an average 26 days, since an extremely low androgen state did not support the tumor growth.

## Discussion

The traditional belief of androgen’s stimulating effect on the growth of PCa originated from Dr. Huggins and Hodges in 1941 [11]. Their study found significant reductions in serum testosterone caused regression of PCa and that increases in testosterone levels enhanced growth of PCa. At that time orchiectomy had demonstrated a dramatic regression in PCa and thus their theory was undoubtedly accepted by urologists and researchers in the field of prostate cancer for numerous decades to follow. Until recent years there has been data suggesting that men with lower serum T levels are more likely to have PCa and T supplementation could offer a protective effect in men with a history of PCa [1–9].

In our previous in vitro study, we demonstrated that the effects of androgen on the proliferation of PCa cells possessed a biphasic pattern, which showed that androgen was essential for the growth of PCa cells, while physiological low levels of androgen were optimal for PCa cell growth, normal and higher levels of androgens inhibited PCa cell growth. This in vivo study confirms that the effect of androgens also possess a biphasic pattern in an animal model. We demonsrated that castrate androgen levels did not support PCa growth, while low levels were optimal for the PCa growth and higher levels of serum testosterone inhibited PCa growth.

Since the PCa LNCaP cell line was established by Dr. Horoszewicz in 1980, it has been considered to represent a useful tool to explore the mechanism of sex hormone action on cell proliferation in an “in culture-in animal” model [12]. The literature published by Dr. Horoszewicz in 1983 described a very detailed characterization on LNCaP cells, including the results of tumor incidence rata and tumor growth rate study on nude mice [13]. When reporting our results, it is useful to compare our data with that reported by Dr. Horoszewicz. It was noticed that in Dr. Horoszewicz’s study, the castrate group had extremely low serum T levels and both the control group and castrated +2 mg T group had low serum T levels mimicking human hypogonadal testosterone levels. There was not a group with higher T levels mimicking human eugonadal testosterone levels. On the basis of their results, it was shown that the groups with lower levels of T had a higher tumor incidence rate than that in castrated group. Our study results demonstrated similar findings. However there was no comparison between groups mimicking higher T levels (i.e. 5 mg T arm) and groups mimicking lower T levels (i.e. 2 mg T arm) in Dr. Horoszewicz’s study. Our study included animals with physiologically low and normal testosterone levels and found that the group with normal T levels for certain period of time had a lower tumor PCa incidence rate than that in group with low T levels. (Table 1).

**Table 1:** Comparison of our tumor incidence rate study with Horoszewicz’s

Horoszewicz’s study groups	Our study arms	Serum T ng/ml	Tumor incidence rate
A. control		1.90	62% (18/29)
	1. Control	1.00	50% (25/50)
B. Castrated		<0.10	22% (7/32)
	2. Castrated	0.10	8% (4/50)
C. Castrated +2 mg T		0.45	57% (8/14)
	3. Normal +2 mg T	2.4 for 2 wks (see Fig. 1)	44% (22/50)
	4. Normal +5 mg T	2.4 for 5 wks (see Fig. 1)	24% (12/50)

It is known that the adult men’s serum androgen level decreases along with age and the prevalence of low-serum testosterone in aging men is projected to be up to 25% [14]. In addition, the incidence rate of PCa increases along with age, as the median age at diagnosis is 66 years old (US National Cancer Institute, 2014). Considering the potential higher risk of PCa in men with lower serum androgen levels, the fact that PCa incidence rates increase and serum androgen level decrease with age, our study offers a possible explanation for these findings. It was reported the ratio of occult PCa in old men (>50 years old) is 2 to 4 times more than clinically diagnosed [15, 16]. We think that clinically diagnosed PCa patients have their serum androgen levels decline more rapidly to lower T levels. Men with occult PCa may have relatively higher serum androgen levels which may better protect against PCa growth.

In addition to surgery and radiation therapy, hormone therapy has been widely used in the treatment of prostate cancer. Current hormone therapy, also defined as androgen deprivation therapy (ADT) or androgen suppression therapy, includes treatments to lower androgen levels by orchiectomy or by using luteinizing hormone-releasing hormone (LHRH) analogs and treatments to inhibit androgen receptor activation by using anti-androgen drugs. Many side effects are associated with ADT, which include sexual dysfunction, hot flashes, osteoporosis, anemia, decreased mental acuity, loss of muscle mass, weight gain, fatigue and depression. These side effects often cause the patients’ life quality to decline dramatically. On the basis of our study, the effect of androgens on PCa growth possesses a biphasic pattern, which suggests that strategies to reduce PCa growth should also consider potentially increase T levels into the normal range by testosterone therapy (TTh).

## Conclusion

The results of this study indicated that the relationship of androgens and PCa growth possessed a biphasic pattern in animals. Castrate T levels were not sufficient to support PCa growth, low T levels were optimal for PCa growth, and higher T levels inhibited PCa growth.

## Abbreviations

ADT: Androgen deprivation therapy
PCa: Prostate cancer
T: Testosterone
TTh: Testosterone therapy
